# Goal-directed mechanisms that constrain retrieval predict subsequent memory for new “foil” information

**DOI:** 10.1016/j.neuropsychologia.2016.07.016

**Published:** 2016-08

**Authors:** David A. Vogelsang, Heidi M. Bonnici, Zara M. Bergström, Charan Ranganath, Jon S. Simons

**Affiliations:** a Department of Psychology, University of Cambridge, Downing Street, Cambridge CB2 3EB, UK; b Behavioural and Clinical Neuroscience Institute, University of Cambridge, Downing Street, CB2 3EB, UK; c School of Psychology, Keynes College, University of Kent, UK; d Center for Neuroscience, University of California at Davis, CA 95618, USA; e Department of Psychology, University of California at Davis, CA 95616, USA

**Keywords:** Episodic retrieval, fMRI, Subsequent memory, foils, left inferior frontal gyrus

## Abstract

To remember a previous event, it is often helpful to use goal-directed control processes to constrain what comes to mind during retrieval. Behavioral studies have demonstrated that incidental learning of new “foil” words in a recognition test is superior if the participant is trying to remember studied items that were semantically encoded compared to items that were non-semantically encoded. Here, we applied subsequent memory analysis to fMRI data to understand the neural mechanisms underlying the “foil effect”. Participants encoded information during deep semantic and shallow non-semantic tasks and were tested in a subsequent blocked memory task to examine how orienting retrieval towards different types of information influences the incidental encoding of new words presented as foils during the memory test phase. To assess memory for foils, participants performed a further surprise old/new recognition test involving foil words that were encountered during the previous memory test blocks as well as completely new words. Subsequent memory effects, distinguishing successful versus unsuccessful incidental encoding of foils, were observed in regions that included the left inferior frontal gyrus and posterior parietal cortex. The left inferior frontal gyrus exhibited disproportionately larger subsequent memory effects for semantic than non-semantic foils, and significant overlap in activity during semantic, but not non-semantic, initial encoding and foil encoding. The results suggest that orienting retrieval towards different types of foils involves re-implementing the neurocognitive processes that were involved during initial encoding.

## Highlights

• We studied brain areas involved in constraining what comes to mind during retrieval.
• Incidental encoding of semantic versus non-semantic new “foil” words was compared.
• Left inferior frontal gyrus exhibited subsequent memory effect for semantic foils.
• Neural overlap between initial processing of semantic information and foil encoding.
• These results suggest sought after information is used to guide recognition memory.

## 1 Introduction

The ability to encode new information into long-term memory is a key feature of human cognition. The brain mechanisms of successful encoding are often studied using ‘subsequent memory’ paradigms, in which neural activation associated with items that are later remembered is contrasted with activation for items that are later forgotten (Paller and Wagner, 2002). Previous fMRI studies have highlighted several regions that are involved in successful episodic encoding, such as the left inferior frontal gyrus (LIFG), medial temporal lobe (hippocampus and parahippocampal region), and posterior parietal cortex (Wagner et al., 1998, Otten and Rugg, 2001, Staresina and Davachi, 2006, Otten, 2007; for reviews see Blumenfeld and Ranganath, 2007, Uncapher and Wagner, 2009, Kim, 2011).

Studies of successful encoding often utilize levels-of-processing (LOP) manipulations, whereby an elaborate encoding task, such as a semantic judgment (e.g. “Is this word pleasant?”), is compared to a less elaborate encoding task, such as non-semantic processing (e.g. “does this word contain the letter O or U?”). The typical finding in these experiments is that the semantic encoding task leads to better retention compared to the non-semantic task, an effect that is observed for intentional encoding, in which participants know that their memory will be tested later, as well as incidental encoding, in which participants are not expecting a memory test to follow (Craik and Lockhart, 1972). Previous fMRI research has shown that the LIFG is associated with the LOP effect, playing an important role in successful encoding of semantic information in particular (Wagner et al., 1998, Gabrieli et al., 1998, Poldrack et al., 1999, Otten and Rugg, 2001, Amunts et al., 2004, Kim, 2011).

Long-term memory does not only depend on the processes that are engaged during encoding, however, but also on processes involved during retrieval. Previous research suggests that retrieval success varies according to the degree of overlap between the cognitive processes engaged during encoding and retrieval experiences (Tulving and Thomson, 1973, Morris et al., 1977, Rugg et al., 2008). Morris et al. (1977), for instance, demonstrated that when retrieval operations were similar to those engaged during encoding (e.g. material was studied in a rhyme phase and was tested with a rhyme retrieval cue) performance was better compared to when participants oriented their retrieval towards a type of information that did not overlap with the operations that were engaged during encoding (e.g. material was studied in rhyme phase and was tested with a semantic retrieval cue). This finding, amongst others, can be accounted for within the Transfer Appropriate Processing framework (Morris et al., 1977, Roediger et al., 1989, Rugg et al., 2008), which states that retrieval success depends upon the extent to which cognitive operations that are engaged during encoding are also involved during retrieval.

An important implication of the transfer-appropriate processing principle is that optimal encoding of a stimulus event depends largely on how memory for the event is tested. This suggests that memory retrieval involves re-implementing the neurocognitive processes that were engaged during encoding and therefore each retrieval attempt likely also involves some degree of encoding (Stark and Okado, 2003). Previous studies have shown that new “foil” words presented amongst studied words during an old/new recognition memory test are better encoded when memory is tested in a semantic memory block compared to a non-semantic memory block (Jacoby et al., 2005a). This result implies that participants invoke a predominantly semantic, and thus deeper, processing mode when attempting to retrieve words encoded during a semantic task and a mainly non-semantic, and thus shallower, processing mode when attempting to retrieve words encoded during a non-semantic task, resulting in better subsequent memory for foils when participants attempt to retrieve semantic information (Jacoby et al., 2005a, Jacoby et al., 2005b; see also Marsh et al., 2009, Danckert et al., 2011, Bergström et al., 2015; for similar findings concerning the “foil effect”).

Previous studies have identified regions in the left frontal cortex (Buckner et al., 2001) and medial temporal lobe regions, such as the hippocampus (Stark and Okado, 2003), as being involved in the incidental encoding of new information. Furthermore, a number of previous studies have investigated the foil effect behaviorally, but to our knowledge the hypothesis that attempting to retrieve semantic versus non-semantic information involves constraining memory retrieval and thereby re-implementing the neurocognitive processes that were engaged during initial encoding has not been tested directly. Here, we applied subsequent memory analysis to fMRI data to investigate how attempting to retrieve semantically versus non-semantically encoded information influences the incidental encoding of new “foil” information. Specifically, we tested the hypothesis that if constraining retrieval towards different types of information involves re-implementing study processes, then subsequent memory effects observed with fMRI for semantic foils should resemble activity patterns observed during the initial semantic encoding task, likely involving regions typically associated with semantic encoding, such as in the LIFG, an effect that should be reduced for incidental encoding occurring via predominantly non-semantic processing. In an adaptation of the foil paradigm developed by Jacoby et al. (2005a), participants made a semantic judgment (“Is this word pleasant or not?”) for words in one block and a non-semantic judgment (“Is there a letter O/U in word?”) for words in another block (phase 1 of the experiment). Next, they performed a blocked old/new memory test, which allowed us to manipulate whether participants oriented retrieval primarily towards semantic or non-semantic information (phase 2). Of special interest were new words (“foils”) that were presented during each memory test block. The semantic foils (new words from the semantic memory test) and non-semantic foils (new words from the non-semantic memory test) were intermixed with completely new words in a final foil recognition test that assessed the ability to discriminate semantic foils, non-semantic foils and new items (phase 3).

We first examined fMRI activity associated with retrieval success during phase 2 as a verification to ensure that our task elicited the typical old/new effects that are reported in previous fMRI literature (McDermott et al., 2009). To identify the neural mechanisms underlying successful encoding of both semantic and non-semantic foils, we then contrasted activation from the first memory test (phase 2) for semantically and non-semantically encoded foils that were subsequently remembered versus forgotten in the final foil recognition test (phase 3). Our main aim was to examine subsequent memory effects that occur while participants constrain memory retrieval for different kinds of information, thereby testing the hypothesis that participants largely re-implement the neurocognitive processes that were engaged during initial encoding of semantic versus non-semantic information. We hypothesized that the LIFG should exhibit greater activation for encoding of semantic foils than non-semantic foils since previous studies have suggested that this region is important for successful encoding of semantic information (Wagner et al., 1998, Gabrieli et al., 1998, Poldrack et al., 1999, Otten and Rugg, 2001, Amunts et al., 2004, Kim, 2011). To test the constrained memory retrieval hypothesis further, a conjunction analysis was conducted to examine the amount of overlap in activation in LIFG between semantic and non-semantic processing during the initial study phase (phase 1) and encoding of semantic and non-semantic foils during the first memory test (phase 2).

## 2 Methods

### 2.1 Participants

Thirty right-handed healthy English native speakers with normal or corrected-to-normal vision participated in this experiment. All participants were screened for MRI scanning and informed consent was obtained before the start of the experiment. Participants received £30 for their participation. The study was approved by the Cambridge Psychology Research Ethics Committee. Data from one participant was excluded because he/she did not finish the task inside the scanner. The data from five participants were excluded because of low trial numbers in some conditions (<12) and data from an additional two participants were excluded because of excessive movement inside the scanner. Thus, the final data set consisted of 22 participants (10 female, 12 male; mean age: 25.7 years, range 18–42).

### 2.2 Materials

The stimuli comprised 552 nouns derived from the MRC psycholinguistic database (Wilson, 1988). The 552 words were split into 6 lists that were matched for concreteness, familiarity, Kucera-Francis Frequency, word length and number of syllables. The assignment of lists to the experimental conditions was counterbalanced across participants.

### 2.3 Procedure

The experiment consisted of three phases that took place in the fMRI scanner: 1) A study phase (henceforth referred to as “phase 1”), 2) A first memory test (“phase 2”), and 3) a foil recognition test (“phase 3”). In phase 1, participants made semantic judgments in one study block (“Is this word pleasant?”) and non-semantic judgments in another block (“Is the letter O or U in the word?”). Each trial started with a randomly jittered 400–600 ms fixation cross, followed by the stimulus that was presented in the center of the screen for 2000 ms. The semantic and non-semantic study blocks consisted of 92 words each, and block order (semantic or non-semantic) was counterbalanced across participants.

In a subsequent blocked test phase (phase 2), participants’ memory was assessed in an old/new recognition test, which manipulated whether participants oriented retrieval predominantly towards semantic or non-semantic information. In the semantic test phase block, old words from the semantic study phase block were intermixed with an equal number of new words (semantic foils). In the non-semantic test phase block, old words from the non-semantic study phase block were intermixed with an equal number of new words (non-semantic foils). The order of test phase blocks (semantic and non-semantic) was counterbalanced across participants, matching the study phase block order. Each test trial began with a randomly jittered 400–600 ms fixation cross, followed by the presentation of the stimulus centrally on the screen for 2000 ms. Importantly, participants were told in advance which memory test they would perform: either the semantic or non-semantic block. For each trial, a cue at the top of the screen reminded the participant during the memory test block which type of information they had to retrieve (“Pleasantness Task” versus “Letter Task”). The trials were pseudorandomized in such a way that no more than three consecutive trials were of the same condition. The semantic and non-semantic test phase blocks consisted of 184 words each (92 old and 92 new “foil” words).

Following the test phase, a surprise foil recognition test (phase 3) was administered in which the semantic and non-semantic foils were intermixed with completely new words. Participants were instructed that they were going to be “presented with a word that was either old or new. ‘Old’ in this case means that you saw the word at some point earlier in the experiment in any study or test phase. ‘New’ words are words you have not seen at all in today's experiment. Press button 1 if you think the word is old and button 2 if you think the word is new”. Phase 3 consisted of 368 words (92 semantic foils, 92 non-semantic foils, and 184 completely new words). Each trial in the foil recognition test began with a 400–600 ms jittered fixation cross followed by the stimulus presented centrally for 2000 ms.

To separate phase 1, phase 2 and phase 3, and to reduce fatigue, participants performed an axis flipping task in which they had to respond with left and right key presses to flip a row of nine “X”s as quickly as possible in a horizontal and vertical orientation for 230 s (see for similar procedures Simons et al., 2006, Gilbert et al., 2010).

### 2.4 fMRI data acquisition

Structural and functional images were acquired with a 3T Siemens TIM Trio system (EPI repetition time [TR]=2000 ms, Echo Time [TE]=30 ms, 32 interleaved axial slices oriented ~10–20° from the AC-PC transverse plane, 3 mm thickness, 1 mm interslice skip, 192 mm field of view [FOV], 64×64 matrix). There were five scanning sessions in total: two study phases (124 volumes each), two test phases (239 volumes each) and one foil recognition test (469 volumes). The first 6 volumes of each session were discarded in order to allow for T1 equilibration. To correct for distortion (Hutton et al., 2002), field maps were acquired with a standard magnetic field mapping sequence (TE=5.19 and 7.65 ms, TR=400 ms, matrix size=64×64) using 32 slices covering the whole head (voxel size 3 mm×3 mm×3 mm).

### 2.5 fMRI analysis

Imaging data were preprocessed and analyzed using SPM8 (Welcome Trust Center for Neuroimaging UCL, London, http://www.fil.ion.ucl.ac.uk/spm). Functional images were realigned with respect to the first to correct for motion, and participants’ structural scans were coregistered to their mean functional image. The coregistered structural scan was segmented to separate out gray matter and generate normalization parameters. Structural and functional images were then normalized into 3×3×3 mm voxels in Montreal Neurological Institute (MNI) stereotactic space (Cocosco et al., 1997). Finally, functional images were spatially smoothed with a 8 mm full-width at half maximum (FWHM) isotropic Gaussian kernel.

For each participant, event types for the conditions of interest were modeled by convolving the onset times of the trials of interest with a canonical hemodynamic response function (see specification of regressors below). Low frequency noise was removed with the use of a 1/128 Hz high pass filter and an AR (1) model corrected for temporal autocorrelation. In order to estimate the parameters for each regressor, a subject-level model was used with movement parameters in the 3 directions of motion and 3 directions of rotation included as vectors of no interest to control for motion confounds.

To investigate the neural activation associated with semantic versus non-semantic processing, we created one General Linear Model (GLM) to analyze the fMRI data collected for phase 1. For this GLM, 2 regressors coded the onsets of 1) all semantic trials presented during the semantic judgment block; 2) all non-semantic trials presented during the non-semantic judgment block. A third regressor coded for trials for which participants gave no response with the purpose of removing noise variability from the first level statistical model. We created two separate GLMs for the fMRI data collected during phase 2: one for retrieval success and one for subsequent memory. The critical difference between these GLMs was that for the retrieval success GLM, new trials were modeled as correct rejections and false alarms based on the participant's response in phase 2, whereas for the subsequent memory GLM, the new trials in phase 2 were modeled as subsequently remembered or forgotten in phase 3. For the retrieval success GLM, 8 regressors coded the onsets of 1) Semantic old trials that were correctly remembered (Semantic hits); 2) Semantic old trials that were not remembered (Semantic misses) 3) Semantic new trials correctly recognized as new (Semantic correct rejections) 4) Semantic new trials recognized as old (Semantic false alarms) 5) Non-semantic old trials that were correctly remembered (Non-semantic hits); 6) Non-semantic old trials that were not remembered (Non-semantic misses) 7) Non-semantic new trials correctly recognized as new (Non-semantic correct rejections) 8) Non-semantic new trials recognized as old (Non-semantic false alarms). A final 9th regressor coded for trials for which participants gave no response.

For the subsequent memory GLM, 4 regressors coded the onsets of 1) Semantic new trials later remembered (“Semantic foils remembered”); 2) Semantic new trials later forgotten (“Semantic foils forgotten”); 3) Non-semantic new trials later remembered (“Non-semantic foils remembered”); 4) Non-semantic new trials later forgotten (“Non-semantic foils forgotten”). A final 5th regressor coded for trials for which participants gave no response.

The analysis focused on two separate ANOVAs: one for retrieval success and one for subsequent memory. For both the retrieval success and subsequent memory factorial analysis, we extracted the mean parameter estimates from *a priori* defined ROIs (see below), and they were entered into two separate ANOVAs (see for similar approaches Flegal et al., 2014, Ritchey et al., 2015). The first ANOVA involved retrieval success (Hits, Correct Rejections)×Experimental condition (Semantic, Non-semantic), and the second ANOVA involved Subsequent Memory (Remembered, Forgotten)×Experimental Condition (Semantic, Non-semantic). To examine the task effects associated with memory retrieval, we focused on regions that are most commonly reported to be involved in recognition success (old/new recognition memory), namely, the bilateral inferior frontal gyrus, bilateral inferior parietal lobe, left precuneus and the posterior cingulate cortex (McDermott et al., 2009). We defined 8 mm spheres around the coordinates provided in a meta-analysis of 18 old/new recognition memory studies by McDermott et al. (2009), which resulted in the following ROIs (transformed from Talairach to MNI space): left frontal cortex ([*x, y, z*]=−43, 22, 24; mean coordinate of the three left frontal coordinates reported in Table 1 from McDermott et al. (2009)), right frontal cortex (31, 27, 34; mean coordinate of the two right frontal coordinates reported in Table 1), left inferior parietal lobe (−36, −66, 37), right inferior parietal lobe (38, −57, 41), left precuneus (−9, −77, 30), and the left posterior cingulate cortex (−5, −46, 28).

For planned analyses looking into subsequent memory effects for both types of foils, we defined 8 mm spherical ROIs centered around the coordinates defined by a recent meta-analysis of 74 subsequent memory studies (Kim, 2011), transformed from Talairach to MNI space. The ROIs included the left inferior frontal gyrus (−46, 26, 19), bilateral hippocampus (left hippocampus: −22, −9, −20; right hippocampus: 18, −7, −19), and bilateral posterior parietal cortex (PPC; left PPC: −28, −80, 35; right PPC: 26, −66, 47). Numerous fMRI studies have reported that these regions play an important role in successful encoding (e.g. Wagner et al., 1998, Kim, 2011). Mean parameter estimates for successful encoding of foils for each of these ROIs were entered in a factorial ANOVA with the factors conditions (Semantic, Non-semantic) and subsequent memory (Remembered, Forgotten).

## 3 Results

### 3.1 Behavioral results

The behavioral results from phase 2 are displayed in Table 1. Recognition accuracy for the first memory test was calculated by the discrimination measure p(Hit)-p(False Alarm) (Snodgrass and Corwin, 1988). Data from phase 2 of one participant was excluded because of a technical error with the data collection during the non-semantic test block, so the statistical analysis of phase 2 was conducted on 21 participants. Planned comparisons confirmed that participants were much better at recognizing semantic compared to non-semantic items (t(20)=16.1, p<0.001, 95% CI [0.46, 0.60], Cohen's Dz=3.5). Furthermore, RTs were faster for old semantic items compared to old non-semantic items (t(20)=4.5, p<0.001, 95% CI [43, 117], Dz=0.99). Foils presented in the semantic condition were also rejected significantly more quickly compared to foils presented in the non-semantic condition (t(20)=3.0, p=0.007, 95% CI [17, 96], Dz=0.65).

The behavioral data from the final foil recognition test (phase 3) are presented in Table 2. Data from 22 participants were included in this analysis. Consistent with prior research, recognition memory (proportion correct) of semantic foils was significantly better than non-semantic foils (t(21)=3.5, p=0.002, 95% CI [0.035, 0.13], Dz=0.75). Accuracy for completely new items in the foil test was significantly higher than for semantic foils (t(21)=2.34, p=0.029, 95% CI [0.014, 0.23], Dz=0.50), and non-semantic foils (t(21)=3.88, p=0.001, 95% CI [0.10, 0.32], Dz=0.83). Participants were faster at recognizing semantic foils compared to non-semantic foils (t(21)=2.84, p=0.01, 95% CI [5.4, 35], Dz=0.60). There was no significant difference in reaction time between both types of foils and new items. These results are a direct replication of earlier findings of the “foil effect” (Jacoby et al., 2005a, Jacoby et al., 2005b, Marsh et al., 2009, Bergström et al., 2015).

### 3.2 fMRI results

#### 3.2.1 Retrieval success

**Fig. 1:**
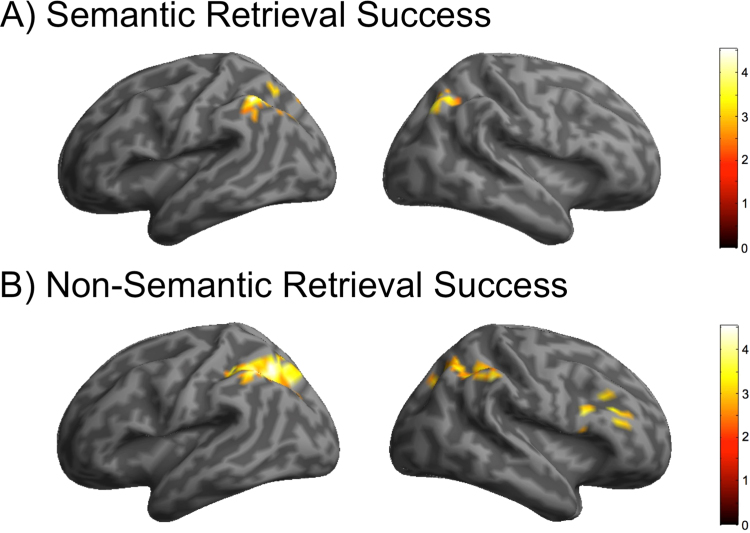
fMRI results for ROI analysis of successful retrieval of semantic (A) and non-semantic (B) information.

fMRI retrieval success effects are reported for 21 participants because of a technical error with the data collection during the non-semantic test block. To examine the neural correlates of successfully retrieving words encoded during semantic versus non-semantic study tasks for each ROI defined by McDermott et al. (2009), mean parameters estimates were entered into a Retrieval Success (Hits, Correct Rejections)×Experimental condition (Semantic, Non-semantic) factorial ANOVA. The results are presented in Fig. 1. The main effect of retrieval success was significant in the left inferior parietal lobe (F(1,20)=9.14, p=0.007, η^2^=0.31) and the right inferior parietal lobe (F(1,20)=12.26, p=0.002, η^2^=0.38), but not in the other ROIs derived from McDermott et al.’s (2009) meta-analysis. There were no significant interactions between condition and retrieval success, suggesting that the inferior parietal lobe is involved in memory retrieval processes regardless of the type of information being targeted.

#### 3.2.2 Subsequent memory

**Fig. 2:**
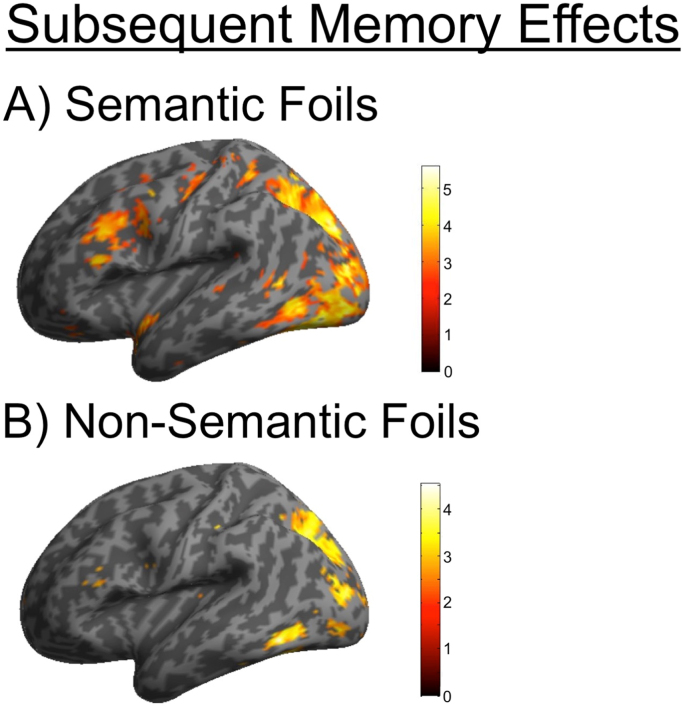
Inflated brain activations of subsequent memory effects.

To investigate the neural correlates of successful encoding of semantic and non-semantic foils, mean parameter estimates for the subsequent memory ROIs defined by Kim (2011) were entered into an Experimental Condition (Semantic, Non-semantic)×Subsequent Memory (Remembered, Forgotten) factorial ANOVA. The regions showing subsequent memory effects for remembered versus forgotten foils are illustrated in Fig. 2. A significant main effect of subsequent memory success was observed in the LIFG (F(1,21)=12.04, p=0.002, η^2^=0.36), left PPC (F(1,21)=9.08, p=0.007, η^2^=0.30), and right PPC (F(1,21)=19.09, p<0.001, η^2^=0.48).

**Fig. 3:**
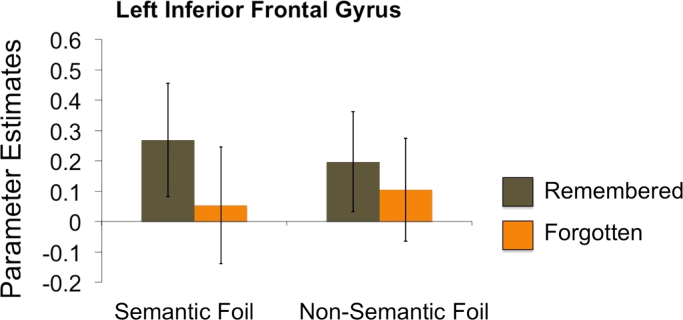
Parameter estimates extracted from the LIFG subsequent memory ROI for both semantic and non-semantic foils that are subsequently remembered and forgotten in phase 3, illustrating the significant subsequent memory by foil condition interaction.

The regional specificity of subsequent memory effects for foils was found to be dependent on the type of information that participants oriented towards. A significant interaction between subsequent memory and condition was observed in the LIFG (F(1,21)=4.54, p=0.045, η^2^=0.18), which was driven by a larger subsequent memory effect for semantic foils (t(21)=3.6, p=0.0016, Dz=0.77) compared to non-semantic foils (t(21)=2.0, p=0.053, Dz=0.43) (See Fig. 3). No significant activations outside the a priori regions of interest survived appropriate statistical correction (FWE p<0.05, whole-brain corrected).

### 3.3 Conjunction analysis

The subsequent memory results imply that participants re-implemented the neurocognitive processes that were originally involved during the encoding tasks in phase 1 whilst attempting to retrieve predominantly semantic versus non-semantic information in phase 2. In order to verify whether the initial encoding during phase 1 and the encoding of foils during phase 2 revealed common activation within the LIFG, we conducted a conjunction analysis to examine the amount of overlap in LIFG activation during phase 1 and phase 2. Conjunction analysis enables comparison of the t-statistic over two contrasts thereby preserving only those voxels that are significantly above threshold in both contrasts (Price and Friston, 1997). Because there was an insufficient number of trials to allow a subsequent memory analysis on phase 1 versus phase 2 data, all trials from phase 1 were included in the conjunction analysis, examining activity associated with the semantic and non-semantic conditions separately. For the semantic condition, we investigated the conjunction between semantic>non-semantic processing from phase 1 and semantic foil remembered>semantic foil forgotten from phase 2. For the non-semantic condition, we examined the conjunction between non-semantic>semantic processing from phase 1 and non-semantic foil remembered>non-semantic foil forgotten from phase 2. Small volume correction was applied using an 8 mm sphere around the LIFG coordinate derived from Kim (2011).

**Fig. 4:**
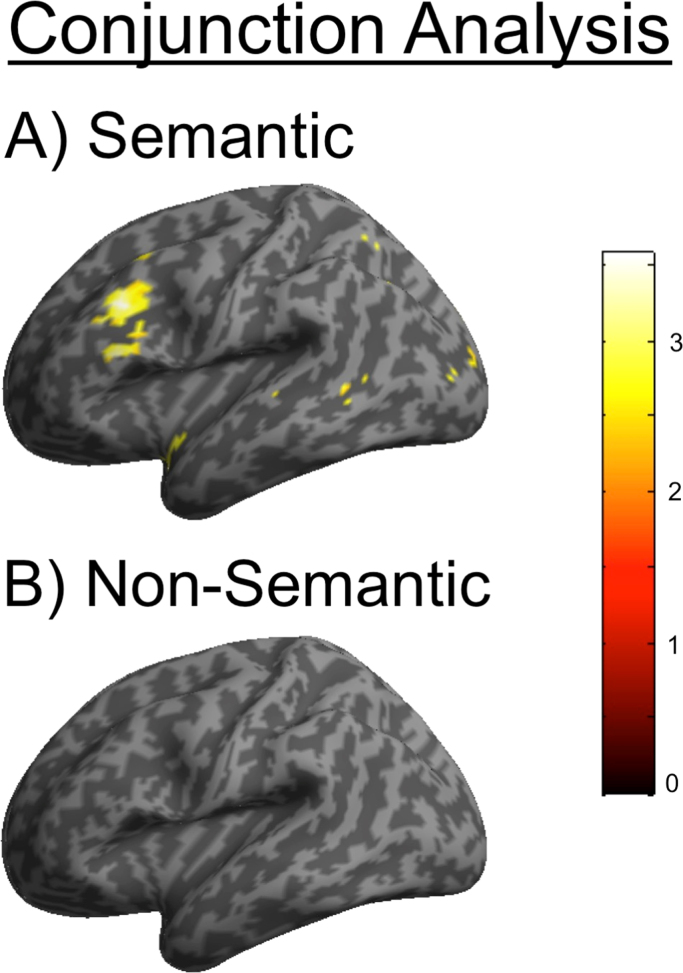
Inflated brain activations of conjunction analysis for phase 1 and phase 2 activity in the LIFG for semantic (A) and non-semantic (B) processing and foil encoding.

The results of the conjunction analysis are displayed in Fig. 4. For the semantic conjunction analysis, significant overlap in activation in the LIFG was observed between phase 1 and phase 2, z=3.33 (peak: −48, 29 19), p(FWE corrected)=0.027. No significant overlap in activation in the LIFG was observed for the non-semantic conjunction analysis, z=−0.32 (peak: −42, 29, 25), p(FWE corrected)=0.67. To investigate whether the difference in the amount of neural overlap between the conditions was significant, we extracted the beta values around the 8 mm LIFG sphere and used these parameter estimates to test for an interaction. This analysis revealed that the conjunction between phase 1 and phase 2 activity for the semantic and non-semantic conditions in the LIFG was significantly different (F(1,22)=8.47, p=0.008, η^2^=0.29).

## 4 Discussion

The aim of the present study was to examine the neurocognitive processes that help constrain what comes to mind during retrieval (Jacoby et al., 2005a, 2005b). We predicted that the neural mechanisms underlying semantic versus non-semantic processing would be largely re-implemented when orienting retrieval towards new “foil” information that was also presented during a memory test phase. Using a priori defined ROIs, we observed significant subsequent memory effects for semantic and non-semantic foils in the LIFG and bilateral PPC. An interesting pattern of activity was discerned in the LIFG (BA 45), which showed a disproportionately larger subsequent memory effect for semantic compared to non-semantic foils. Conjunction analysis revealed significant overlap in the LIFG between semantic processing in phase 1 and encoding of semantic foils in phase 2, but not between non-semantic processing and encoding of non-semantic foils. The findings that the subsequent memory effect for semantic foils in the LIFG resembled typical semantic encoding activity (Kapur et al., 1994; Wagner et al., 1998, Gabrieli et al., 1998; Poldrack et al., 1999; Otten and Rugg, 2001; Fletcher et al., 2003, Blumenfeld and Ranganath, 2007; Kim, 2011), and that there was overlap in activation between semantic processing in phase 1 and semantic foil encoding in phase 2, suggest that orienting retrieval towards different types of foils largely involves re-implementing the neurocognitive processes that were engaged during encoding.

Additional evidence for a difference in the processing of semantic versus non-semantic foils came from the behavioral results, which indicated that recognition memory performance during phase 3 was significantly better for semantic foils than non-semantic foils, despite the fact that these two types of foils only differed with respect to the type of information participants had oriented towards when they were first encountered during phase 2 (Jacoby et al., 2005a, 2005b). Turning to the brain mechanisms involved in successful encoding of foils, we observed significant subsequent memory effects for foils in the LIFG and bilateral posterior parietal cortex. These regions have all been implicated in successful encoding in previous studies (Wagner et al., 1998; Davachi et al., 2001; Simons and Spiers, 2003; Blumenfeld and Ranganath, 2007; Kim, 2011), and the LIFG has been previously linked with encoding of foils (Buckner et al., 2001). However, the current results go further by finding that the LIFG was disproportionately engaged during encoding of subsequently remembered semantic compared to non-semantic foils, which is in line with previous research that has indicated a role for the LIFG in semantic compared to non-semantic processing (Wagner et al., 1998; Poldrack et al., 1999; Devlin et al., 2003; Amunts et al., 2004). Patients with LIFG lesions exhibit impairments in tasks that involve controlled retrieval of semantic information (Swick and Knight, 1996; Thompson-Schill et al., 1998; Martin and Cheng, 2006). Furthermore, transcranial magnetic stimulation over LIFG has been found to impair semantic processing but leave non-semantic processing intact (Gough et al., 2005), and to disrupt later memory performance for semantic information (Machizawa et al., 2010).

The conjunction analysis provided evidence for the neurocognitive re-implementation account by revealing that there was significant overlap in LIFG activation between semantic processing in phase 1 and encoding of semantic foils in phase 2, thereby supporting the view that incidental encoding of foils may involve intentionally re-enacting the encoding task from phase 1 to facilitate memory retrieval. An alternative explanation is that participants may adopt a retrieval orientation that allocates attention to a deeper level of analysis of the words, which may lead to deeper levels of processing of the foil words. Together with the behavioral findings, these neuroimaging results suggest that sought after information is used to guide information processing during recognition memory (Jacoby et al., 2005a). Jacoby et al. (2005a) have referred to this as source constrained retrieval, which is related to the assumption that successful memory retrieval is dependent in large part upon reinstated context, i.e. a match between the encoding context and retrieval context. This view is similar to the Transfer Appropriate Processing Framework, which states that successful retrieval depends on the amount of overlap between encoding and retrieval processes (Morris et al., 1977). The current behavioral and neuroimaging findings provide converging evidence for the idea that neurocognitive processes implemented during encoding can be re-implemented during a memory test phase. That is, cue specification during phase 2 served to constrain memory search and influenced the incidental encoding of old items and foils, resulting in better subsequent memory for foils when they had been predominantly processed semantically, as opposed to non-semantically. In this way, performance on a memory task can be considered to be largely dependent on the extent to which cognitive modes at test are similar to the cognitive modes engaged during initial encoding and therefore each retrieval attempt involves some degree of encoding as well (Stark and Okado, 2003).

The other region exhibiting a significant subsequent memory effect for foils in the current data, namely the PPC, may play a slightly different role in the encoding process. Subsequent memory effects in the superior parietal lobe are thought to reflect task general goal-directed processes that are necessary for item selection and attention (Corbetta et al., 2008; Uncapher and Wagner, 2009; Kim, 2011). These attentional processes play an important role in encoding relevant information into long term memory and likely also recruit frontal regions as part of the frontoparietal attentional network (Vincent et al., 2008; Kim, 2011). The hippocampus is also a region that has been associated with subsequent memory effects (e.g. Simons and Spiers (2003)). In the current data the hippocampus showed only a non-significant trend effect for successful encoding of foils, but previous research has implicated the hippocampus as a region playing a role in successful encoding by binding content representations into a coherent long-term memory representation (Paller and Wagner, 2002; Kim, 2011). Moreover, a previous study found that hippocampal activity during successful incidental encoding of new items presented in a memory test phase resembled the encoding related activity during intentional learning, thereby suggesting that incidental and intentional successful encoding may rely on similar regions within the hippocampus (Stark and Okado, 2003).

The current study indicates that people can constrain their memory search for the specific type of information they are looking for and that the LIFG in particular is involved in strategic processes that orient retrieval towards the type of information being targeted. Although there has previously been little investigation of the brain mechanisms involved in the incidental encoding of different types of foils, several studies have investigated the “foil effect” behaviorally (Jacoby et al., 2005a, 2005b; Marsh et al., 2009; Danckert et al., 2011; Halamish et al., 2012; Alban and Kelley, 2012; Kantner and Lindsay, 2013; Bergström et al., 2015). Marsh et al. (2009), for example, examined whether recognition memory for deep versus shallow foils during the final foil recognition test was driven by recollection, the ability to remember specific details and contextual information from an event, or familiarity, the feeling of knowing that an item had encountered before without recollecting any specific details from that encounter (Yonelinas, 2002). Marsh et al. found that recognition memory for semantic items during the final foil test was related to more “remember” responses compared to non-semantic foil recognition, indicating that orienting retrieval towards deep semantic as opposed to shallow non-semantic foils yields more qualitative differences in recollection rather than familiarity. Bergström et al. (2015) extended the foil effect to self-referential source recollection and showed that even subtle differences between retrieval conditions, in this case conceptual versus agentic self-referential recollection, can reveal differences in recognition memory for conceptual versus agentic foils assessed in a later foil recognition test.

Another line of research has focused on how constraining retrieval is related to the amount of cognitive control that is required during retrieval. Velanova et al. (2003) used a variation of the foil paradigm in which they showed that foils presented in a high cognitive control condition were retained better than foils presented in a low cognitive control condition. Their neuroimaging results revealed that the LIFG was more responsive in the high relative to the low cognitive control condition, suggesting that LIFG activation is dependent upon the level of cognitive control that is required for memory retrieval. Consistent with these findings, Alban and Kelley (2012) showed that the foil effect is reduced if the first memory test (during which the foils are “encoded”) is preceded by a recognition test that does not require a high level of cognitive control. This result implies that performing an initial easy recognition test induces less effortful retrieval processes that are “carried over” to the memory test phase in which foils are encoded, resulting in diminution of the “foil effect” (Alban and Kelley, 2012). Furthermore, older adults may not exhibit a significant “foil effect” (Jacoby et al., 2005b), perhaps attributable to inefficient engagement of cognitive control processes supported by the prefrontal cortex that help constrain memory search during the early stages of retrieval (Velanova et al., 2007). Previous studies have suggested that the LIFG is not a functionally homogenous region, instead consisting of distinct sub-regions that process semantic or non-semantic information (Poldrack et al., 1999; Dobbins et al., 2002). The extent to which different levels of cognitive control relate to constrained retrieval of semantic versus non-semantic information and map onto the functional and anatomical architecture of the LIFG and its sub-regions is a matter future studies should investigate further.

To conclude, our findings provide a characterization of the neurocognitive mechanisms underlying strategic retrieval processes, demonstrating a differentiation between encoding of semantic versus non-semantic foils by pointing towards a role for the LIFG, in particular, in the encoding of semantic foils. This finding indicates that sought after information is used during processes supported by the LIFG, and other regions, to guide information processing during recognition. Our results provide novel insights into the mechanisms underlying successful memory encoding by demonstrating that constraining retrieval to focus on semantic versus non-semantic information involves the re-implementation of study processes during the act of remembering.

## Conflict of interest

None.

## Figures and Tables

**Table 1:** Hits-False Alarms (FA) and reaction time (RT) for phase 2.

	Hits-FA		RT (ms)			
	Mean	SD	Hits (Mean)	Hits (SD)	Correct rejections (Mean)	Correct rejections (SD)
Semantic	0.75	0.12	1057	112	1084	127
Non-semantic	0.22	0.11	1137	137	1141	148

**Table 2:** Accuracy (proportion correct) and RT for phase 3.

	Accuracy		RT	
	Mean	SD	Mean	SD
Semantic foils	0.68	0.16	1030	122
Non-semantic foils	0.60	0.17	1051	129
New items	0.80	0.11	1050	133
